# The complete chloroplast genome of Korean bred apple ‘Kamhong’ (*Malus domestica* Borkh.)

**DOI:** 10.1080/23802359.2022.2139159

**Published:** 2022-11-11

**Authors:** Se Hee Kim, Kang Hee Cho, Sang-Yun Cho, Byeong Hyeon Yun

**Affiliations:** Fruit Research Division, National Institute of Horticultural and Herbal Science, Wanju, Korea

**Keywords:** Apple, Korean cultivar, Kamhong, chloroplast genome

## Abstract

Apple (*Malus domestica* (Suckow) Borkh., 1803) is economically important horticultural fruit crop in the world. In this study, the complete chloroplast genome of Korean apple cultivar ‘Kamhong’ was characterized through the *de novo* assembly of Illumina sequencing data. The chloroplast genome is a circular molecule of 161,069 bp length with 36.55% GC content and has a total of 112 genes including 78 protein-coding genes, 30 transfer RNA genes, and 4 ribosomal RNA genes. Phylogenetic analysis with protein-coding sequences of chloroplast genome revealed that ‘Kamhong’ was closely grouped with *M. domestica* cultivars reported in China. The genomic data generated in this study can extend a molecular basis for phylogenetic relationships of Korean cultivar ‘Kamhong’ with other *M. domestica* cultivars bred in other countries.

Korean apple (*Malus domestica* (Suckow) Borkh., 1803) cultivar ‘Kamhong’ was bred by crossing between ‘Spur EarliBlaze’ and ‘Spur Golden Delicious’ and become a popular fruit crop in Korea due to its high yield and sugar content (Shin et al. 1996). ‘Kamhong’ was cultivated in the experimental field of National Institute of Horticultural and Herbal Science (35°50´42″N, 127°8´51″E) and its Voucher specimen (IT249255) was registered at National Agrobiodiversity Center in Korea (http://genebank.rda.go.kr/eng/uat/uia/actionMain.do, Mun-sup Yoon, msyoon63@korea.kr). Although there are many reports chloroplast genomes of *M. domestica*, most of them were from Chinese cultivars and one chloroplast genome was reported from South Africa when searched in GenBank (accessed in 21 September 2022). Therefore, the chloroplast genome of the Korean cultivar ‘Kamhong’ is the first reported sequence information in Korea, and this is thought to be important for studying the phylogenetic relationship and breeding history of apple cultivars.

Genomic DNA was extracted from leaves using DNeasy Plant Mini kit (QIAGEN, Germany). Illumina paired-end (PE) library was constructed and sequenced using the Illumina HiSeqX platform with 151-bp PE reads. Raw sequencing data of 2.1 Gb were trimmed using quality_trim program in CLC Assembly Cell package ver. 4.2.1 (QIAGEN, Denmark) and used for *de novo* assembly of chloroplast genome according to previous study (Kim et al. 2015). In brief, trimmed high-quality read sequences were *de novo* assembled using clc_novo_assemble program in the CLC Assembly Cell and then chloroplast contigs were selected and ordered by similarity searches with reported *Malus* chloroplast genomes (MK434922.1, MK434917.1, and LT996898.1). The selected contigs were merged and gap-filled to generate complete chloroplast genome. Sequence error in the genome was checked and corrected by read mapping and manual curation. Genes were annotated using the GeSeq (Tillich et al. 2017) and Artemis (Carver et al. 2012) programs with reported *Malus* chloroplast genomes and manually curated to determine. Precise gene regions using BLAST searches against reported *Malus* chloroplast genomes.

Chloroplast genome of ‘Kamhong’ is a circular molecule of 161,069 bp length with 36.55% GC content (GenBank accession number MZ647488), which is slightly longer than the already reported chloroplast genome of ‘Golden delicious’ and ‘Yantai Fuji 8′ (Yan et al. 2019). The genome consists of a pair of inverted repeats (IRs) of 26,352 bp, a large single copy (LSC) of 88,184 bp and a small single copy (SSC) of 19,181 bp. A total of 112 genes were predicted in the genome including 78 protein coding genes, 30 transfer RNA genes and 4 ribosomal RNA genes.

The complete chloroplast genome sequences of other *Malus* species were used to construct the phylogenetic tree and *Pyrus pyrifolia* (Burm.f.) Nakai, 1926 as an outgroup. Phylogenetic analysis of ‘Kamhong’ with other *Malus* species was performed using a Maximum-Likelihood (ML) method with conserved protein-coding sequences and revealed that ‘Kamhong’ was closely grouped with *M. domestica* cultivars reported in China (Figure 1). The complete chloroplast genome sequence will provide useful genetic information for understanding the phylogenetic relationships of Korean cultivar ‘Kamhong’ with other M. domestica cultivars bred in other countries.

**Figure 1.**
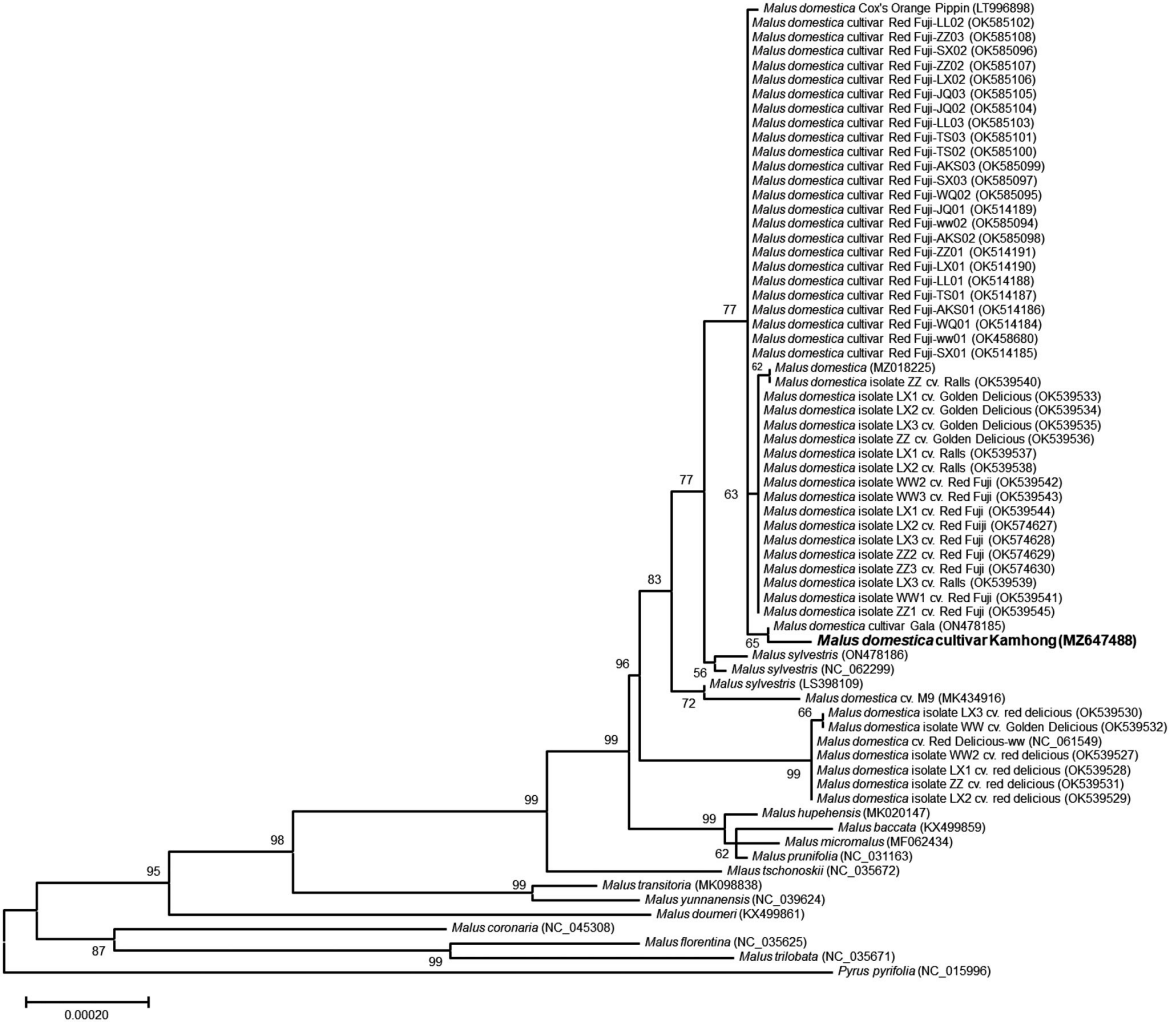
Maximum-Likelihood phylogenetic tree of chloroplast genomes of ‘Kamhong’ and its related species. A total of 70 protein-coding sequences conserved in the chloroplast genomes of 68 species were multiple-aligned using MAFFT (http://mafft.cbrc.jp/alignment/server/index.html) and used to generate phylogenetic tree by MEGA 11.0 (Tamura et al. 2021). The bootstrap support values (>50%) from 1000 replicates are indicated on the nodes. GenBank accession nos. of chloroplast genome sequences used for this tree are indicated within parentheses.

## Data Availability

The genome sequence data that support the findings of this study are openly available in GenBank of NCBI at https://www.ncbi.nlm.nih.gov under the accession no. MZ647488. The associated BioProject, Bio-Sample and SRA numbers are PRJNA749119, SAMN20353434 and SRR15219603, respectively.
